# Prescribing indicators at primary health care centers within the WHO African region: a systematic analysis (1995–2015)

**DOI:** 10.1186/s12889-016-3428-8

**Published:** 2016-08-22

**Authors:** Richard Ofori-Asenso, Petra Brhlikova, Allyson M. Pollock

**Affiliations:** 1 Research Unit, Health Policy Consult, P. O. Box WJ 537, Weija-Accra, Ghana; 2 Centre for Primary Care and Public Health, Barts and the London School of Medicine & Dentistry, Queen Mary, University of London, Yvonne Carter Building, 58 Turner Street, London, E1 2AB UK

**Keywords:** Prescribing indicators, Drug use indicators, Pharmacoepidemiology, Prescribing evaluation, Medicine utilization studies, Systematic reviews, Africa

## Abstract

**Background:**

Rational medicine use is essential to optimize quality of healthcare delivery and resource utilization. We aim to conduct a systematic review of changes in prescribing patterns in the WHO African region and comparison with WHO indicators in two time periods 1995–2005 and 2006–2015.

**Methods:**

Systematic searches were conducted in PubMed, Scopus, Web of science, Africa-Wide Nipad, Africa Journals Online (AJOL), Google scholar and International Network for Rational Use of Drugs (INRUD) Bibliography databases to identify primary studies reporting prescribing indicators at primary healthcare centres (PHCs) in Africa. This was supplemented by a manual search of retrieved references. We assessed the quality of studies using a 14-point scoring system modified from the Downs and Black checklist with inclusions of recommendations in the WHO guidelines.

**Results:**

Forty-three studies conducted in 11 African countries were included in the overall analysis. These studies presented prescribing indicators based on a total 141,323 patient encounters across 572 primary care facilities. The results of prescribing indicators were determined as follows; average number of medicines prescribed per patient encounter = 3.1 (IQR 2.3–4.8), percentage of medicines prescribed by generic name =68.0 % (IQR 55.4–80.3), Percentage of encounters with antibiotic prescribed =46.8 % (IQR 33.7–62.8), percentage of encounters with injection prescribed =25.0 % (IQR 18.7–39.5) and the percentage of medicines prescribed from essential medicines list =88.0 % (IQR 76.3–94.1). Prescribing indicators were generally worse in private compared with public facilities. Analysis of prescribing across two time points 1995–2005 and 2006–2015 showed no consistent trends.

**Conclusions:**

Prescribing indicators for the African region deviate significantly from the WHO reference targets. Increased collaborative efforts are urgently needed to improve medicine prescribing practices in Africa with the aim of enhancing the optimal utilization of scarce resources and averting negative health consequences.

## Background

According to the World Health Organization (WHO), more than half of all medicines are inappropriately prescribed, dispensed or sold with such practices deemed to be most prevalent in healthcare settings in the developing world where mechanisms for routine monitoring of medicines use are still in early stages of development [1–4]. In developing and low middle income countries, pharmaceuticals account for a high proportion of household and overall healthcare expenditure [5]. Improvement in the way in which medicines are used is important in reducing morbidity and mortality, building public confidence, reinforcing health system credibility as well as optimising the utilisation of scarce resources [6–8]. The “wise list” in Stockholm, Sweden for instance, is an example of an improvement in medicine use with an essential medicines list (EML) with high adherence to just 200 medicines to improve physician familiarity with quality medicines and reduce costs in a high income country that could provide valuable lessons for developing countries seeking to optimize resource utilization [9].

Since the late 80's, the WHO together with the International Network for Rational Use of Drugs (INRUD) have been advocating proper documentation of medicines use and have developed core drug use indicators in three related areas of prescribing practices, patient care and facility specific factors [10]. The drug use indicators are regarded as objective measures that can be extended to describe patterns of medicines usage in any health facility, country or an entire region.

The core drug use indicators include five prescribing indicators which are meant to detail particular prescribing characteristics related to poly-pharmacy, antibiotic use, injection use, generic prescribing and adherence to the essential medicines list (EML) [10, 11]. Even though an international standard of the prescribing indicators has not been empirically determined, the WHO has recommended reference values for each of the indicators (see Table 1) [12, 13]. In 1993, the WHO published the guideline “How to investigate drug use at health facilities: selected drug use indicators” aimed at outlining methods for the collection and presentation of information on medicines use in primary health care (PHC) settings [10]. Subsequently, the WHO has been publishing information on global medicines usage as part of its World Medicines Situation reports [1, 4]. A more detailed fact book focusing mainly on medicines use at PHCs in developing and transitional countries was also published in 2009 [14]. The broadest review on medicines usage was published in 2013; this incorporates data from 900 studies covering facilities at various level of care in 104 countries between 1990 and 2009 [15]. For the African region, the review reported the average number of medicines per patient encounter to be 2.6, percentage of encounters with antibiotics prescribed as 45.9 %, percentage of encounters resulting in prescription of injection as 28.4 %, percentage of medicines prescribed from EML to be 89.0 % and percentage of medicines prescribed in generic name as 65.1 % [15]. Despite not meeting the WHO targets, the estimates show relatively frequent prescribing from EML and of generic products. The high percentage of antibiotic and injection prescriptions has been attributed to disease burden, weak health systems and patients’ preferences. A trend analysis showed ‘little progress over time’ [15].

**Table 1:** WHO prescribing indicators and recommended reference values [12, 13]

WHO prescribing indicator	Reference value
Average number of medicines per encounter	<2
Percentage of medicines prescribed by generic name	100 %
Percentage of encounters with an antibiotic prescribed	<30 %
Percentage of encounters with an injection prescribed	<20 %
Percentage of medicines prescribed from an essential medicines list or formulary	100 %

The WHO African Region is one of the six regions of the WHO and consists of 47 member states with over 927 million inhabitants in 2013 [16]. The region faces one of the greatest disease burden compared to all other WHO regions. In 2013, the life expectancy at birth in Africa was 58 years, the lowest among all the WHO regions and 10 years below that of Southeast Asia (68 years), the region with the second lowest life expectancy [16]. According to the 2013 Global burden of disease estimates, while their relative burdens have seen some decline, communicable, newborn, nutritional, and maternal causes such as diarrhoeal diseases, lower respiratory infections, and protein-energy malnutrition still remain the top drivers of health loss in most African countries [17]. Yet, many countries in the region are also experiencing significant epidemiological transition characterised by a growing burden of non-communicable diseases (NCDs) thereby resulting in a "double disease burden" [18]. For instance, a recent systematic review demonstrated consistent increase in prevalence of hypertension in Africa from 19.7 % in 1990 to 27.4 % in 2000 and 30.8 % in 2010 [19]. While the emerging double disease burden presents unique public health challenges and may call for greater intervention measures resources for improving health delivery in Africa remain scarce. In 2013, the region’s average total health expenditure per capita (PPP int. $) was 222, the lowest among all WHO regions and extremely low when compared to Europe (2214) and Americas (3873) [16]. Additionally, there are siginficant gaps in available health system structures that hinder effective healthcare delivery. For instance, in the period 2007–2013, the physician to population ratio (per 10,000 population) in Africa was 2.7; this was far lower than the global average of 13.9 [16]. According to Motie [20], financial and human resource challenges have hindered many healthcare systems within the African region from evolving to meet the emerging healthcare demands. The increasing emergence of non-communicable diseases is likely to further exacerbate these trends.

Most health systems in Africa do not have established mechanisms for routine system-wide medicine monitoring and utilization. Moreover, reviews of specifically designed studies are deemed to be out of date after 3 to 5 years or even less (Whitlock et al.) [21]. This paper presents a systematic review to summarize available information on prescribing indicators for the WHO African region over the last two decades (1995–2015). Our aim was to critically appraise the quality of studies on prescribing practices in the Africa region and to compare the results of studies on prescribing indicators at PHCs in the African region against WHO recommended reference values. We also wished to understand whether there are observable differences in prescribing at private and public facilities in the WHO African region. To this end, we defined public to represent all fully government owned or quasi-governmental facilities. Private was defined to cover for-profit and mission health facilities.

## Methods

### WHO prescribing indicators

The prescribing indicators measure the performance of healthcare providers in five key areas related to the appropriate use of medicines (Table 1) [10]. The derivation of these indicators for any health facility (s) is based on an analysis of patient clinical encounters. A patient encounter is recognized to represent “the duration of interaction between patient and health provider. Ideally, this encounter includes a number of components: history taking, diagnosis process: selection of non-pharmacological or pharmacological treatment, prescription (and perhaps dispensing) of treatment; and explanations about treatment and its adverse effects, follow-up, and prevention.” [22]. The encounters may be analyzed retrospectively using data from medical history records or can be analyzed prospectively as patients arrive during the period of data collection [10]. It is important to highlight that the determination of the core prescribing indicators does not require information on patients’ signs and symptoms as they provide general prescribing tendencies (non-disease specific). The various prescribing indicators are meant to elucidate peculiar prescribing characteristics relating to polypharmacy, level of antibiotic and injection use and adherence to guidelines relating to generic and EML prescribing [23].

### Studies retrieval process

We conducted a structured review of the literature in accordance with the PRISMA (Preferred Reporting Items for Systematic Reviews and Meta-Analyses) guidelines [24]. Comprehensive searches were conducted in PubMed, Scopus, Web of science, Africa-Wide Nipad, Africa Journals Online (AJOL), Google scholar and INRUD Bibliography databases. The main key words used were “primary health care, primary health services, community health centres, community-based healthcare, health facilities, primary healthcare settings’ AND “prescribing indicators, prescribing patterns, drug use indicators, drug utilization patterns, prescribing evaluation, prescribing statistics, rational prescribing, rational use of medicines, health facility indicators” AND “Africa, Sub-Saharan Africa, WHO African Region”. The main limits used were ‘humans’ and ‘English’. Additionally, we searched references of published reviews and selected papers for additional publications.

### Inclusion and exclusion of studies

We included only observational studies published in English in peer-reviewed Journals between 1^st^ January 1995 and 31^st^ December 2015, which reported at least one WHO/INRUD core prescribing indicator or where these indicators were derivable from results/data presented. A study must have specified the total number of patient encounters involved for it to be accepted into the review. Furthermore, to minimise potential bias, studies in which the patient encounters were derived through a random sampling technique were mainly included [25]. For studies with duplicate publications, the version published first or one with complete dataset was selected. In the case of interventional studies, we included only pre (baseline) values. Although, most hospital facilities provide secondary level care, in certain instances, outpatient departments provide primary care services. Hence, where full description of this has been provided, studies conducted in such settings were included.

### Critical appraisal of studies

Each study’s quality was assessed using a 14-point scoring system modified from the Downs and Black checklist with inclusions of recommendations in the WHO guidelines (Table 2) [10, 26]. We awarded a one point value if study satisfied each criteria. If study did not meet criteria, it was awarded a zero. As studies may not assess all the five indicators (e.g. a study may not measure antibiotic use), the criteria were applied in relation to the indicator (s) assessed. In view of this, the quality grading was expressed as a percentage. Irrespective of the number of criteria applied, a study is considered as ‘high quality’ if it scores ≥70 % of the total tally scores based on the applicable criteria. A score of 69–51 % was regarded as ‘moderate quality’ and a score of ≤50 % was graded as ‘low quality’.

**Table 2:** Studies’ quality appraisal checklist [10, 26]

1. Objective of the study clearly described 2. Study design or data collection methods clearly stated 3. Participants representative of a general patient population (Ideally studies of prescribing indicators should involve a sample of general illness encounters representing a mix of health problems) 4. Adequate sample size (WHO recommends a minimum of 600 encounters) 5. Whether type of facility was specified (i.e. public or private) 6. Whether the number of facilities involved was specified 7. Patient age/gender and other characteristics reported 8. Whether study described how medicines were counted (WHO recommends that FDCs should be counted as one) 9. Whether study defined the medicines to be regarded as antibiotic according to the WHO/INRUD classification if antibiotic indicator was assessed (Only affects quality in terms of % antibiotics use) 10. Whether the reference essential medicines list (EML) used in the study was specified. Researchers may utilize the WHO model EML, facility EML or national EML as reference guide 11. Whether study specified the medicines regarded as injections. Ideally, routine immunizations should not be counted as injections (Only affects quality in terms of % of injections use). 12. Whether the statistical method employed in analyzing the results of the study was appropriate and fully described 13. Whether the study described how missing data was handled and if any confounders 14. Whether the study results were discussed appropriately. For instance, if conclusion (s) were relevant to the findings	

### Statistical analysis

Due to the wide heterogeneity of studies, a formal meta-analysis was not conducted. We therefore adopted a more descriptive approach as employed in previous reviews [14, 15]. For each WHO/INRUD prescribing indicator, we determined the median as well as the 25 and 75^th^ percentiles [14]. Mean values of prescribing indicators across studies was not used as this would be unduly influenced by outliers [27]. To minimise the influence of larger sample-sized studies, prescribing indicator values were not weighted by sample size [14]. In this case, the approach we adopted was to treat each study as a single data point with equal weight, without regard to sample size and variance. All computations were done electronically using Microsoft Excel 2015® and results of prescribing indicators were compared to the WHO’s recommended reference values and with previous reported values [12–15]. Statistical estimates of the difference between the results of prescribing indicators obtained for private and public PHCs as well as between different publication periods was not conducted since variance would have been greatly underestimated in such circumstances [14]. Sub-analysis was also conducted across different facility ownerships (private vs public) as well as across the studies publication periods 1995–2005 and 2006–2015.

## Results

### Studies identification and retrieval

**Fig. 1:**
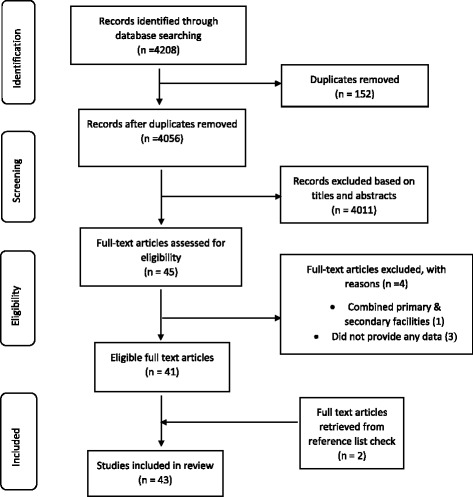
Schematic flow diagram of studies' search and retrieval steps

Figure 1 outlines the schematic flow of the studies' identification and inclusion processes. A total of 4208 articles were identified by literature search. After the exclusion of duplicates and irrelevant studies based on titles and abstracts, 45 articles were retrieved for detailed full-text analysis. Out of the 45 studies, 41 met the inclusion criteria for addition to the review. Two (2) additional studies were identified through the reference screening bringing the total number of studies included in the review to forty-three (43) [7, 8, 22, 28–67]. The 43 studies included in this review (Table 3) collectively reported WHO/INRUD prescribing indicators based on overall analysis of 141,323 patient encounters across 572 PHCs. The PHCs included 359 (62.8 %) public and 213 (37.2 %) private facilities. We were unable to separate ‘mission-based’ and ‘business/for-profit’ in the private facilities category as studies gave a limited description of their activities. About 65.1 % (*n* = 28) of studies were published in the period 2006–2015 whereas 34.9 % (*n* = 15) were published in the years 1995–2005. The 43 studies included in this review were conducted in 11 countries representing 23.4 % (11/47) of countries in the region under study. The 11 countries included Ghana (4), Nigeria (11), Tanzania (6), Kenya (1), Gambia (1), Zambia (1), Zimbabwe (2), South Africa (6), Ethiopia (7), Burkina Faso (2) and Botswana (2).

**Table 3:** Descriptive characteristics of included studies

No.	Author Details	Year of Publication	Country	Data collection method	Data collection duration	Mean age of patients	Type of facility (s)	No. of facilities	Setting	Prescriber type (s)	No. of patient encounters
1.	Abdella and Wabe [28].	2012	Ethiopia	Retrospective	1 year	n.s	Public	1	Urban	Health Officers	384
2.	Adisa et al. [29].	2015	Nigeria	Prospective	3 months	≥15	Public	8	Urban	n.s	400
3.	Afriyie and Tetteh [30].	2013	Ghana	Retrospective	7 months	n/s	Public	1	Urban	Medical assistants, Doctors	120
4.	Afriyie et al. [31].	2015	Ghana	Prospective	6 months	n/s	Public	1	Urban	Medical assistants, doctors	3127
5.	Ahiabu et al. [32].	2015	Ghana	Retrospective	1 year	n/s	Private & Public	4 (public = 1, private = 3)	Urban	Doctors, medical assistants	1600
6.	Angamo et al. [33].	2011	Ethiopia	Prospective	6 weeks	n/s	Public	4	Urban	n.s	3058
7.	Babalola et al. [8].	2011	Nigeria	Retrospective	1 year	19.4 years	Public	20	n/s	Community health workers, physicians, health assistants, pharmacy technicians, nurses and Pharmacist	560
8.	Ball et al. [34]	2000	Zimbabwe	Prospective	n/s	n/s	Public	1	Urban	Nurses	31
9.	Bantie [35]	2014	Ethiopia	Prospective	n/s	n/s	Public	6	n/s	n.s	600
10.	Bexell et al. [36]	1996	Zambia	Retrospective	3 months	n/s	Public	8	Urban	Clinical officers	1167
11.	Boonstra et al. [37]	2002	Botswana	Prospective	n/s	25.5	Public	30	Rural & Urban	Registered nurse, Family nurse practitioner	2994
12.	Boonstra et al. [38]	2005	Botswana	Prospective	n.s	<5	Public	30	Rural & Urban	Nurses	255
13.	Bosu and Ofori-Adjei [39]	2000	Ghana	Retrospective	1 year	15 years	Public	6	Rural	Doctors and medical assistants	585
14.	Desta et al. [40]	1997	Ethiopia	Prospective & Retrospective	Prospective 1–2 days Retrospective-1 year	n/s	Public	18	n/s	n.s	2340
15.	Dippenar et al. [41]	2006	South Africa	Prospective	2 month	n/s	Public	1	Urban	Doctors, nurses	1000
16.	Enato et al. [42]	2012	Nigeria	Retrospective	6 months	n/s	Public	1	Rural	Doctors, nurses	315
17.	Enato et al. [43]	2013	Nigeria	Retrospective	1 year	n/s	Public	3	Urban	Community health officers	1440
18.	Isah. [44]	2008	Nigeria	Retrospective	1 year	n/s	Private	20	Rural	n.s	2510
19.	Kapp et al. [45]	2013	South Africa	Retrospective	3 months	41.0 years	Public	4	Urban	n.s	400
20.	Katende-Kyenda et al. [46]	2007	South Africa	Retrospective	1 year	n.s	private	9	Urban	n.s	83,655
21.	Krause et al. [47]	1999	Burkina Faso	Prospective	2 months	n/s	Public	9	Rural	nurses	313
22.	Massele and Nsimba. [48]	1997	Tanzania	Prospective	20 days	n/s	Public & Private	40 (public = 20, private = 20)	Urban	n.s	1200
23.	Massele et al. [49]	2001	Tanzania	Retrospective	14 months	n/s	Private	20	Rural & Urban	Doctors	1200
24.	Massele et al. [50]	2007	Tanzania	Retrospective	1 year	n/s	Public & Private	20 (public = 10, Private = 10)	n/s	n.s	2000
25.	Massele et al. [51]	2012	Tanzania	Prospective	3 months	n/s	Public & Private	20 (public = 10, private = 10)	Urban	Clinical officers, and other paramedics	2000
26.	Meyer et al. [52]	2001	South Africa	Retrospective	1 month	n/s	Public	22	n/s	Nurses	1287
27.	Mohlala et al. [53]	2010	South Africa	Prospective	5 months	n/s	Private	36	Rural & Urban	Doctors	276
28.	Nsimba et al. [54]	2004	Tanzania	Retrospective	1 year	n/s	Private	10	n/s	n.s	600
29.	Nsimba [55]	2006	Tanzania	Prospective	n/s	<5 years	Public	10	n/s	n.s	652
30.	Odusanya and Oyediran [7]	2000	Nigeria	Retrospective	6 months	n/s	Public	12	n/s	Community health officers, Public health nurses	650
31.	Olayemi et al. [56]	2006	Nigeria	Prospective & Retrospective	Prospective-2–3days Retrospective-n/s	n/s	Public	20	Rural & Urban	n.s	1560
32.	Oyeyemi and Ogunleye [57]	2013	Nigeria	Retrospective	1 year	34 years	Public	4	Urban	Medical officer, community health officer, nurses and community health extension workers	600
33.	Phillips-Howard et al. [58]	2003	Kenya	Retrospective	1 year	<5	Public	8	Rural	n.s	9318
34.	Risk et al. [59]	2013	Gambia	Retrospective	1 year	1.71	Public	20	Rural & Urban	n.s	2400
35.	Savadogo et al. [60]	2014	Burkina Faso	Prospective	1 month	<5	Public	2	Urban	n.s	376
36.	Shiferaw et al. [61]	2010	Ethiopia	Retrospective	1 year	n/s	Public	19	n/s	n.s	731
37.	Sisay and Mekonnen [62]	2012	Ethiopia	Retrospective	2 years	n.s	Public	2	Urban	n.s	424
38.	Suleman et al. [63]	2013	Nigeria	Retrospective	3 months	n/s	Public	10	n/s	n.s	222
39.	Tamuno [64]	2011	Nigeria	Retrospective	1 year	n.s	Private	10	Urban	n.s	998
40.	Trap et al. [22]	2002	Zimbabwe	Retrospective	<6 months	n/s	Private	57	Urban	Doctors	1699
41.	Truter et al. [65]	2010	South Africa	Retrospective	8 weeks	n/s	Public	1^*m*^	Rural	Supervised student trainees	4026
42.	Tsega et al. [66]	2012	Ethiopia	Retrospective	1 year	25 years	Public & Private	11 (public = 3, private = 8)	Rural & Urban	n.s	600
43.	Uzochukwu et al. [67]	2002	Nigeria	Retrospective	n/s	n/s	Public	33	n/s	n.s	1650

*n.s* not specified, *m* mobile clinic

### Quality of studies

Overall, using the quality assessment criteria outlined, 51 % of studies were graded as of high quality whereas 42 % and 7 % were graded as of medium and low quality, respectively. The major factors that affected quality grades of studies included smaller sample size, lack of adherence to WHO recommendations (especially counting and classification of medications) and poor reporting of study information. Around one-third (32.6 %) of studies included in the review involved patient encounters <600 and were deemed to be small per recommendations outlined in the WHO guidelines [10]. This is an important consideration as studies with larger sample size are more likely to present representative/generalizable results.

The studies collected data either prospectively (using current patients as they present for consultation) or retrospectively (using past medical records). In 27 studies, data on prescribing were collected retrospectively, in 14 studies this was done prospectively while another two studies used a mix approach of collecting prescribing information both prospectively and retrospectively. The fact that majority of studies adopted a retrospective approach is quite understandable as such data are easier to collect. Nonetheless, retrospective analysis introduces some bias if certain information is excluded owing to poor record keeping. In the study by Babalola et al. [8] in Nigeria for instance, records of 40 patients were excluded from the analysis because they had incomplete data while in the case of Massele et al. [50] in Tanzania, the patient registers of three consecutive years were abandoned for another register because they had incomplete data. It is possible that the excluded information may have presented different prescribing characteristics than those reported in the studies. Also most retrospective analyses rely on prescription sheets and hence may exclude patients who are not prescribed medicines. This is likely to lead to overestimation of variables such as average number of medicines per patient, injection prescribing rate and antibiotic prescribing rate although EML and generic prescribing rates are unlikely to be affected. While studies that adopted a prospective approach may minimize the loss of data and deal with other limitations of retrospective assessments, they also introduce an observer bias (Hawthorne effect) as it is difficult to blind the health facility staff. In the Nsimba et al. [55] study in Tanzania for instance, all health staff were briefed on the study prior to prospective data collection. Prescribers may modify their behaviour if they know they are been investigated and as such, results derived this way may also not be representative of typical prescribing behaviour [10]. In the two studies that adopted dual prospective and retrospective analysis, no significant difference in results were observed in the two approaches thereby affirming to a large extent the validity of their findings [40, 56].

It is recommended that prescribing indicators are analysed over an extended period (ideally ≥1 year) to minimize the impact of seasonal variations in morbidity patterns, peculiarities in staffing and inconsistencies in medicines supply which can all impact on the patterns of medicines prescribing [10, 39]. However, across studies reviewed, the period over which prescribing data were collected varied widely from as short as 1 day to as long as 24 months. Nineteen studies reported data collection period less than 1 year and these are likely to be prone to seasonal variations in prescribing and may not necessarily represent usual trends.

### Average number of medicines prescribed per patient encounter

Information on the number of medicines prescribed per patient encounter was obtained from 40 studies that included a total 138,671 patient encounters. Among these studies, the median number of medicines prescribed per patient encounter was 3.1 (IQR 2.3–4.8) (Table 4). The average number of medicines prescribed per patient encounter was higher for public 2.6 (IQR 2.2–4.7) than private 2.5 (IQR 2.3–3.2) centres. The reported average medicines prescribed per patient was higher for studies published in the period 2006–2015 (3.5; IQR 2.2–5.6) than the period 1995–2005 (2.4; IQR 2.3–4.0).

**Table 4:** Summary of prescribing indicators at PHCs within the WHO African region

	Prescribing indicators				
	Average number of medicines prescribed per encounter^a^	Percentage of medicines prescribed by generic name	Percentage of encounters^b^ with an antibiotic prescribed	Percentage of encounters^b^ with an injection prescribed	Percentage of medicines prescribed from an essential medicines list
WHO reference values [12, 13]	<2	100 %	<30 %	<20 %	100 %
*Facility type*					
All	3.1 (IQR 2.3–4.8)	68.0 % (IQR 55.4–80.3)	46.8 % (IQR 33.7–62.8)	25.0 % (IQR 18.7–39.5)	88.0 % (IQR 76.3–94.1)
	n = 138,671	n = 121,797	n = 120,422	n = 40,096	n = 33,140
Public^a^	2.6 (IQR 2.2–4.7)	68.9 % (IQR 57.6–84.5)	45.0 % (IQR 30.1–60.2)	25.6 % (IQR 14.1–4.8)	89.9 % (82.9–95.6)
	n = 44,596	n = 28,046	n = 26,071	n = 28,400	n = 23,044
Private^a^	2.5 (IQR 2.3–3.2)	61.3 % (IQR 47.7–75.7)	51.3 % (IQR 37.5–66.6)	29.0 % (IQR 19.0–39.5)	84.0 % (IQR 69.8–91.9)
	n = 92,475	n = 92,151	n = 92,751	n = 10,096	n = 8496
*Studies publication period*					
1995–2005	2.4 (IQR 2.3–4.0)	64.2 % (IQR 51.9–77.9)	43.1 % (IQR 33.7–61.7)	25.0 % (IQR 17.1–41.4)	87.1 % (IQR 84.9–92.0)
	n = 25,289	n = 13,949	n = 15,971	n = 14,549	n = 10,324
2006–2015	3.5 (IQR 2.2–5.6)	70.4 % (IQR 60.7-81.1)	49.0 % (IQR 37.8–63.1)	24.8 % (IQR 18.7–37.4)	88.9 % (IQR 70.8–94.0)
	n = 113,382	n = 107,848	n = 104,451	n = 25,547	n = 22,816

*IQR* interquartile range, *n* total number of patient encounters used in analysis, ^a^excludes Ahiabu et al. [32] which did not provide individual results for public and private facilities

### Percentage of medicines prescribed by generic name

Generic prescribing rate was reported in 33 studies that involved a total of 121,797 patient encounters. Among these studies, the generic prescribing rate was 68.0 % (IQR 55.4–80.3). Public PHCs reported a higher percentage (68.9 %; IQR 57.6–84.5) of medicines prescribed generically than private centres (61.3 %; IQR 47.7–75.7). Generic prescribing rate for studies published in the period 2006–2015 (70.4 %; IQR 60.7–81.1) was higher than for studies published in the period 1995–2005 (64.2 %; IQR 51.9–77.9).

### Percentage of encounters with antibiotic prescribed

Data on antibiotic prescribing rate was also retrieved from 34 studies comprising of a total of 120,422 patient encounters. The overall proportion of encounters resulting in the use of antibiotics was 46.8 % (IQR 33.7–62.8). Public PHCs reported lower antibiotic prescribing rate (45.0 %; IQR 30.13–60.2) compared to private facilities (51.3 %; IQR 37.5–66.6). Higher antibiotic prescribing rate was recorded among studies published in the period 2006–2015 (49.0 %; IQR 37.8–63.1) than for those published in the period 1995–2005 (43.1 %, IQR 33.7–61.7).

### Percentage of encounters with injection prescribed

Injection prescribing rate was retrieved from 32 studies consisting of a total 40,096 patient encounters. The overall proportion of encounters resulting in the prescription of an injection was 25.0 % (IQR 18.7–39.5). The proportion of encounters at public PHCs which resulted in the prescription of an injection was determined as 25.6 % (IQR 14.1–44.8) while that of private facilities was 29.0 % (IQR 19.0–39.5). Injection prescribing rate across studies published in the period 2006–2015 (25.0 %; IQR 17.1–41.4) was similar to studies published in the period 1995–2005 (24.8 %; IQR 18.7–37.4).

### Percentage of medicines prescribed from an essential medicines list or formulary

Adherence to EML was determined using data from 27 studies in which a total of 101,077 medicines were prescribed.  The overall proportion of medicines prescribed from an EML was estimated as 88.0 % (IQR 76.3–94.1). Higher proportion of prescriptions from public centres (89.9 %, IQR 82.9–95.6) adhered to the use of EML than private centres (84.0 %; IQR 69.8–91.9). EML use rate was higher among studies published in the period 2006–2015 (88.9 %; IQR 70.8–94.0) than for the studies published within 1995–2005 (87.1 %; IQR 84.9–92.0).

## Discussion

### Average number of medicines per patient encounter

Our review showed a high number of medicines (3.1) prescribed per patient encounter. This value is higher than that reported by the WHO factbook for the African region (2.6) and that for the European (2.5), Southeast Asia (2.5) and the Americas (1.8) regions [14]. The WHO analysis was however based on a larger number of studies as the review was not limited to studies published in peer-reviewed journals, but included those reported in NGOs and ministry of health reports as well as from other grey literature. On the other hand, while the WHO factbook and other reports have generally reported higher number of medicines prescribed per patient in private compared to public facilities we found the reverse with slightly higher number of medicines per patient in public (2.6) than private (2.5).

A generally high number of medicines prescribed per patient exceeding WHO reference value may point to polypharmacy as an increasing problem in Africa. Many parts of the region are experiencing a changing epidemiological transition creating a double disease burden of both communicable and NCDs [68] and there is evidence that poly-pharmacy becomes more prominent when health personnel need to treat multiple diseases simultaneously [69, 70]. Additionally, demographic shifts in most parts of Africa is resulting in an increasing elderly population who are likely to suffer significant co-morbidities and need for multiple medications [71, 72]. In countries like Ghana, Kenya, Nigeria and Tanzania the population aged 60 and over is projected to increase by around 147 %, 144 % and 80 %, respectively between 2005 and 2030 [73]. Such patterns may partly account for the observed higher number of medicines prescribed per patient in the period 2006–2015 (3.5) as compared to the period 1995–2005 (2.4). Nonetheless, a number of studies reviewed reported very high levels of symptomatic management of cases [44, 50], and this may have also contributed to the overall high number of medicines prescribed per patient.

Excessive use of multiple medicines per patient (poly-pharmacy) is likely to result in increased risk of adverse drug interactions, dispensing errors and decreased patients’ knowledge of the correct doses of medications. In the study in Nigeria by Uzochukwu et al. [67], the percentage of patients remembering their dosing schedules decreased significantly as the number of medicines increased whereas Kapp et al. [45], reported a direct correlation between the number of medicines prescribed and the occurrence of adverse events in South Africa. Increased risk of drug adverse effects as a result of  poly-pharmacy could create a cycle of health demands and costs as new treatments may be required [74].

### Percentage of medicines prescribed by generic name Generics

The generic prescribing rate attained in this study (68.0 %) was lower than that recommended by the WHO (100 %). This result however portrays a better generic prescribing rate than reported by the WHO factbook for the African region (60 %) albeit based on smaller number of studies. However, the results appear lower when compared to values reported for the WHO’s Western Pacific region (78 %), although higher than generic prescribing rates reported for the Eastern Mediterranean (27.7 %) and Southeast Asian regions (48.9 %) [14]. The lower generic prescribing rate observed in private than public centres is consistent with trends reported by the factbook as well as other WHO reports [1, 14, 15].

The overall improved generic prescribing rate as documented by higher generic prescribing for the period 2006–2015 compared to the period 1995–2005 may be due to the increasing availability of standard medicines as generics. For instance, over 45 top brand medications are expected to have patent expired between 2011 and 2020 and thus likely to make generic versions readily available [75]. Once patency expires and availability/accessibility improves, lower cost becomes an incentive that could drive generic prescription. As an example; higher rates of generic prescribing [for proton pump inhibitors (PPIs) and statins] were seen in South Africa in 2010/2011 among patients enrolled into medical aid schemes receiving discounted medications [76]. In Netherlands, similar trends have been observed where about threefold increase in statins utilization was observed between 2000 and 2010 despite a 58 % decrease in reimbursed expenditure mainly as a result of multiple supply and demand measures, including a preferential pricing policy [77]. Furthermore, in recent years, considerable education and studies demonstrating no difference in outcomes between originators and generics across a wide range of products and classes including antipsychotics, anti-infectives and cardiovascular medicines have been undertaken and these may have contributed to the increase in generic prescribing [78–81].

The lower generic prescribing rates observed for private facilities may be due to the fact that prescribers in the private sector may perceive generic medicines as not financially rewarding as patients typically purchase medicines from same facilities and there may be a financial incentive to prescribe most expensive products [22]. Additionally, the more frequent prescribing of innovator (expensive) brands in the private sector may be due to prescriber’s quest to satisfy the expectations of their clients (often the - well -to do) who may falsely perceive the issuance of expensive (innovator) medicines as constituting ‘quality care’. Persistent prescription of branded (innovator) medicines is likely to result in increased treatment costs. In a study by Nwolisa et al. at outpatient centres in Nigeria, the difference in cost between same drugs prescribed in brand names as against generic names were between 41.7 % and 60 % [82]. Nicolosi and Gray investigated the cost impact of generic and proprietary prescribing among chronic disease patients in South Africa and their findings indicated that of “all generic medicines identified 67.5 % were more than 40 % cheaper, per defined daily dose (DDD) per month, than the branded version” [83]. An analysis of facility-based medicines price data from 17 countries by Cameron and Laing [84], found that an average of 9–89 % could be saved by switching from originator brands to lowest-price generic equivalents. To further improve generic prescribing, diverse approaches may be adopted including addressing fears related to generics, thorough education of prescribers (beginning when they are in school or training) or in some instance through the adoption of a compulsory INN prescribing policy [85, 86].

### Percentage of encounters with an antibiotic prescribed

The percentage of encounters with antibiotics prescribed in this review was 46.8 % which exceeds the reference value of <30 % recommended by the WHO [12]. The antibiotic use rate in this study is similar to that reported by the WHO (47 %) [14]. However, it is lower when compared to estimates provided for the Eastern Mediterranean region (53.2 %) but higher than that of the Americas (39.3 %) and European (33.5 %) regions [14]. A higher value for antibiotic prescribing was reported for the private facilities (51.3 %) than public facilities (45 %) which does imply that antibiotic prescribing may be more of a problem in the private than public sector—an observation consistent with WHO reported trends [1, 14].

The higher antibiotic prescribing rate reported for the 2006–2015 period than for the 1995–2005 period may point to a non-improving or potentially worsening problem of antibiotic use in Africa. The overall high levels of antibiotic prescribing may partly be accounted for by the extensively documented high burden of infectious diseases within the African region. For instance, in the studies by Massele et al. [50] in Tanzania, Enato et al. [41] in Nigeria and Bosu and Ofori-Adjei [39] in Ghana, 58 %, 38.3 % and 22 % of conditions presented at the PHCs, respectively were attributable to infectious diseases (excluding malaria). These high levels of reported infections are likely to contribute to more antibiotic presciption. Additionally, in many parts of Africa, HIV/AIDS remains endemic which although does not require the use of antibiotics can increase the prevalence of opportunistic bacterial infections necessitating the use of antibiotics [53].

Regardless of the seemingly high levels of infection rates, not all antibiotic prescribing and use reported may be appropriate. In a number of studies, antibiotics were reported as been prescribed to treat diseases like malaria, diarrhoea and RTIs (mostly viral in origin) conditions which do not usually require antibiotic use [40, 56, 59]. At PHCs in many parts of Africa, microbiology laboratory facilities are often non-existent and as such prescribers may rely mainly on their clinical judgment. While empirical use of antibiotics based on clinical judgment other than laboratory confirmations is permitted in many instances such as otitis, apparent pneumonia and cellulitis, it is well recognized that consistent use of antimicrobials when infection or diagnosis has not been established or fully confirmed can lead to overprescribing [87]. While it is recommended good practice that medicines are written for specified diagnosis, in one study conducted in Nigeria by Isah [44], over 50 % of the patients’ folders reviewed had no established diagnosis. In another study conducted in Ethiopia by Desta et al. [40], the researchers reported that any compliant presented by a patient was recorded as final diagnosis. One study investigated prescribing patterns across different health professionals and found higher level of antibiotic prescribing more prevalent in lower cadre staff like community nurses and health assistants than in medical doctors and pharmacists [8]. However, across all the studies, higher antibiotic use were generally reported for mix of health workers (physicians, nurses, medical assistants etc.). Lack of in-service training was recognised as contributing to poor prescribing practices as demonstrated by one study in Ghana, in which the investigators reported that for the PHCs surveyed, none of the prescribers had received an in-service training in the preceding 5 years [39].

In addition to lack of adequate training, prevailing socio-cultural factors and demand are known to influence irrational antibiotics use [88]. These factors were reported by some studies to have influenced prescribing behaviours [40]. In private settings, prescribers are more likely to adhere to patient demand for antibiotics and injections for fear of losing out on customers and this may underline the higher antibiotic prescribing rate observed. Some studies found a correlation between patient overload and injection and antibiotic use [22, 64]. In many parts of Africa there are widespread reports of acute shortage of health staff, therefore in many instances, health personnel may play dual role of prescriber and dispenser. Such occurrences can be a breeding ground for irrational prescribing as no control mechanisms will be in place to check wrong, incorrect or poor prescribing. Dispensing prescribers may be more likely to prescribe irrationally in the private-for-profit sector where there may be a financial incentive for over-prescribing. For instance, Trap et al. [22] found that PHC dispensing doctors were more likely to prescribe antibiotics than non-dispensing doctors in Zimbabwe. High patient load and inadequate prescriber time can contribute to irrational prescribing as prescribers may find it more convenient and time-saving to prescribe an antibiotic rather than educate a patient that his condition does not require an antibiotic as it will require more lengthy discussion [89]. In a study in Ghana by Polage et al. [90], 98 % of physicians stated that they rarely order or never order tests, because of time constraints.

Indiscriminate use of antibiotics backed by no diagnostic certainty can contribute to the development of drug resistance [87]. In one study included in this review, the researchers carried out further antibiotic sensitivity testing. Their findings indicated that, vaginal and endocervical isolates were always resistant to the commonly used antibiotics such as ampicillin and tetracycline but almost always sensitive to antibiotics like cefuroxime and gentamicin which were less frequently prescribed at the facilities [39]. The development of antibiotic drug resistance can cause significant morbidity and mortality as infectious disease rates remain high in the African region. High use of antibiotics is also costly and the development of resistance can further aggravate treatment cost by requiring the use of more powerful and expensive antibiotics which are likely to be unavailable in many parts of Africa. In the Bosu and Ofori-Adjei study in Ghana, antibiotics alone accounted for about 40 % of treatment cost in patients in whom they were prescribed [39].

### Percentage of encounters with an injection prescribed

The overall injection prescribing rate determined in this study was 25.0 % which exceeds the reference value (<20 %) recommended by the WHO [12]. The WHO fact book reported an injection use rate of 27.5 % which is a bit higher than that attained in this study [14]. The result also indicates a higher use of injectable medications when compared to results reported for Eastern Mediterranean (20.1 %), European (17.2 %) and West Pacific (23.2 %) regions. In comparison, the study found higher use of injections at private facilities (29 %) than at public centres (25.6 %) which is also in accord with global trends reported by the WHO [1, 14]. The similar injection prescribing rate in the periods 2006–2015 and 1995–2005 may highlight a non-changing injection use behaviours among health personnel in Africa.

Widespread injection prescribing was reported across all mix of health workers (doctors, nurses, medical assistants etc.). Patient preference, socio-cultural beliefs have been also noted to influence prescribing behaviours. In a study by Massele and Mwaluko in Tanzania, it was reported that some patients walked into the PHC facility with their own supply of injectable medicines, syringes and needles asking for them to be prescribed these medicines because they believed injections were more powerful in restoring and maintaining health than other formulations [91]. As indicated previously, patient influences are likely to be felt more in the private sector where there may be a financial implication if prescribers do not adhere to client demands. As the administration of injections often requires supervision by skilled health care providers, the frequency of prescription of injectable medications is important [92]. Excessive and indiscriminate use of injections can increase the risk of spreading blood-borne diseases such as hepatitis B and even HIV/AIDS especially in a region where infections rates remain high. Moreover, overuse of injections sets up a cycle of repeated visits putting pressure on healthcare staff and driving costs.

### Percentage of medicines prescribed from an essential medicines list or formulary

The overall EML prescribing adherence of 88.0 % in this study is comparable to the 87.8 % reported by the WHO albeit lower than the optimal recommended value (100 %) [13]. The EML prescribing rate presented in this review is higher when compared to estimates reported for other regions like the European (55.1 %), Americas (71.4 %) and the South East Asia (81 %) regions [14]. The results obtained indicate that adherence to EML when prescribing is better at public (93.5 %) that private facilities (83.95 %), a pattern consistent with what has been reported previously by the WHO [14, 15].

The general high EML prescribing rate may be due to wider adoption of the use of EML in many countries as well as expanding number of medicines on various EMLs [93]. Regardless, the non-optimal use of EML as reported in this study can be attributed to a myriad of factors such as ineffective distribution of EML, inadequate sensitization among health workers and a general lack of enforcement mechanisms. In separate studies by Bosu and Ofori-Adjei [39] and Odusanya and Oyediran [7] conducted in Ghana and Nigeria, respectively, all the facilities studied lacked a copy of an EML. Moreover, some studies reported that the main source of information for prescribers were drug representatives [39, 49]. Such sources have been documented to be problematic as drug companies may over-represent the efficacy of their medicines, discredit the efficacy of competitor brands and likely to induce prescribers to prescribe outside established guidelines [94, 95]. The lower EML adherence observed in private practice may be due to the fact that in many countries in Africa, the private sector is encouraged but not obliged to prescribe from EML as may be the case for public centres [53].

### Limitations

This systematic review has some limitations. Firstly, the identified studies were concentrated in a few (11 out of 47) countries in the studied region. While a lack of research into this area in parts of Africa may have contributed to this; it may have also been due to the inclusion of only articles published in English and also the exclusion of grey literature. Around one-third (32.6 %) of studies included in the review involved patient encounters <600 and were deemed to be small per recommendations outlined in the WHO guidelines [10]. This review also took the assumption that the African region is homogenous, although, in reality, there may be differences in disease burden, health system challenges, socio-cultural and political climates across countries which all can affect how medicines are used. Majority of the studies (74 %) also collected data retrospectively. We consider that retrospective analysis may result in the overestimation of poly-pharmacy (average number of medicines), antibiotic utilization and injection use because patients who were not given a prescription are likely to be excluded [25]. In our analysis we stratified results by key sector (public and private) but did not control for differences in prescriber characteristics. Therefore, the apparent differences in the prescribing indicators between the two sectors may be due to multiple factors. We assessed prescribing indicators at two time points and this is unlikely to reveal much about prescribing trends. Importantly, this review reports indicator-based studies which are unable to ascertain whether the reported prescribed medicines were actually taken by the patients involved. Indicator-based studies while able to identify medicine use problem areas, do not answer the question of rationality or appropriateness of treatment which may require a different methodology and analysis [23]. It is also important to reiterate that while the prescribing indicator reference values are useful, these have not been empirically determined and the extent to which different factors influence them have not been thoroughly investigated [25]. 

## Conclusion

Our analysis reveals prescribing indicators at PHCs within the WHO African region which deviate significantly from proposed reference values. While our review is based on limited studies, it does highlight that some improvements in prescribing practices are needed. The prescribing patterns observed are reflective of population factors as well as varied health system challenges on the African continent. Greater commitments from governments and all stakeholders are required to improve medicine prescribing practices in the region. This is necessary not only to avert negative health consequences but also to afford the optimal utilization of scarce resources.

## Abbreviations

AIDS, acquired immune deficiency syndrome; EML, essential medicines list; GDP, gross domestic product; HIV, human immunodeficiency virus; LRI, lower respiratory infection; NCD, non communicable disease; PHC, primary health care; PRISMA, preferred reporting items for systematic reviews and meta-analyses; RTI, respiratory tract infections; WHO, World Health Organization
